# Fecal microbial characterization of hospitalized patients with suspected infectious diarrhea shows significant dysbiosis

**DOI:** 10.1038/s41598-017-01217-1

**Published:** 2017-04-24

**Authors:** Tzipi Braun, Ayelet Di Segni, Marina BenShoshan, Roy Asaf, James E. Squires, Sarit Farage Barhom, Efrat Glick Saar, Karen Cesarkas, Gill Smollan, Batia Weiss, Sharon Amit, Nathan Keller, Yael Haberman

**Affiliations:** 1 Sheba Medical Center, Tel-HaShomer, 5265601 Israel; 2 Children’s Hospital of Pittsburgh of UPMC, 4401 Penn Ave, Pittsburgh, PA 15224 USA; 3 Tel Aviv University, Ramat Aviv, Tel-Aviv 6997801 Israel; 4 Cincinnati Children’s Hospital Medical Center, 3333 Burnet Ave, Cincinnati, OH 45229 USA

## Abstract

Hospitalized patients are at increased risk for acquiring healthcare-associated infections (HAIs) and inadequate nutrition. The human intestinal microbiota plays vital functions in nutrient supply and protection from pathogens, yet characterization of the microbiota of hospitalized patients is lacking. We used 16S rRNA amplicon sequencing to characterize the global pattern of microbial composition of fecal samples from 196 hospitalized patients with suspected infectious diarrhea in comparison to healthy, non-hospitalized subjects (n = 881), and to traditional culture results. We show that hospitalized patients have a significant rise in α-diversity (richness within sample) from birth to <4 years of age, which continues up to the second decade of life. Additionally, we noted a profoundly significant increase in taxa from Proteobacteria phylum in comparison to healthy subjects. Finally, although more than 60% of hospitalized samples had a greater than 10% abundance of Proteobacteria, there were only 19/196 (10%) positive cultures for *Campylobacter*, *Salmonella*, or *Shigella* entero-pathogens in traditional culturing methods. As hospitalized patients have increased risk for HAIs and inadequate nutrition, our data support the consideration of nutritional and/or microbial modification in this population.

## Introduction

The human intestinal microbiota ecosystem plays an essential role in processing energy and nutrients, protecting cells from injury, and promoting local and systemic immunity. Deviation from what is considered a healthy microbiota, or dysbiosis, may impair vital functions such as nutrient supply, vitamin production, and protection from pathogens. Age^1, 2^, geography^3^, lifestyle^4, 5^, illness^4^, and medications^6^ have all been associated with the gut microbiome composition, but many populations and conditions have not yet been fully characterized. Hospitalized patients are at increased risk for acquiring healthcare-associated infections (HAIs), which are infections that are acquired while receiving medical treatment in healthcare facilities. Gastrointestinal illnesses are the second most frequently reported HAIs^7^ and *Clostridium difficile* is the most common pathogen constituting 12.1% of these potentially devastating gastrointestinal nosocomial infections. Notably, hospitalization alone has been shown to affect the microbiome in a recent study that compared outpatient elderly volunteers to hospitalized elderly patients. This study demonstrated a marked reduction in the *Bacteroides-Prevotella* group in those who were hospitalized and an increase in Enterobacteriaceae, specifically in the hospitalized patients who did not receive antibiotics^8^. Few other studies have sought to characterize the gut microbiome in individuals with, or recovering from, enteric infections^9, 10^ but no study has analyzed hospitalized patients with suspected infectious diarrhea.

Bacterial stool culture is an integral part of the standard clinical microbiological investigation, but is limited by a low yield^11, 12^. Using typical bacterial stool culture methods, enteric infection is conceptualized as a binary state where the pathogen is either present in the gut or not. Newer molecular techniques such as broad-range high throughput 16S rRNA amplicon sequencing (16S-seq) and metagenomics shotgun sequencing can be applied to capture the overall microbial composition of the culturable and non-culturable taxa. These techniques have already had a substantial impact on our understanding of the epidemiology of many diarrhea-associated bacteria including the discovery that horizontal gene exchange facilitated the emergence of a novel, highly virulent strain of Shiga toxin-producing *Escherichia coli* (STEC) during the European outbreak of hemolytic-uremic syndrome in 2011^13^. Other studies, using these investigative modalities, have demonstrated that bacteria currently not considered as important diarrhea-causing pathogens, are positively associated with diarrhea in young children from low-income countries^14^. Here, we use 16S-seq to characterize the global microbial composition of a prospective Israeli non-selected cohort of hospitalized patients suspected to have infectious diarrhea (n = 196). We compare the microbial composition in this population to healthy individuals, and to traditional bacterial culture results. We noted a significantly profound increase in taxa from Proteobacteria phylum in comparison to healthy subjects that could not be explained by identification of entero-pathogens in traditional culturing methods. As hospitalized patients are at increased risk for acquiring HAIs, identifying the dysbiosis associated with this population can serve as a first step toward specific therapeutic interventions in this population. In support of this concept is a recent study that showed that a mixture containing Lactobacillus GG and micronutrients reduced the incidence of HAI^15^.

## Results

### A cross-section prospective study to characterize the microbial composition of hospitalized patients with suspected infectious diarrhea

To study the gut microbiota of hospitalized individuals with suspected infectious diarrhea we prospectively collected stool samples submitted to the Clinical Microbiology Lab at the Sheba Medical Center in Israel between Feb-May 2015. Stool specimens were subjected, in parallel, to conventional microbiological culture performed at the Clinical Microbiology Lab and to broad-range high throughput 16S-seq. Patients’ demographic characteristics are shown in Table 1. DNA was extracted and the V4 region of the 16S gene was sequenced using Illumina MiSeq. The data were analyzed using the QIIME pipeline as outlined in the methods section. Data from healthy individuals were obtained from a healthy adult Israeli cohort^16^ (Israeli healthy 1) and local healthy volunteers’ (Israeli healthy 2) fecal samples were collected and analyzed similarly to the hospitalized samples. Our primary goal was to characterize the microbial composition and diversity of non-selected Israeli hospitalized individuals with suspected infectious diarrhea and to compare the results to healthy individuals. Our secondary goal was to test how 16S-seq results correlates with traditional culture results.

**Table 1:** Demographic characteristics and culture results of Israeli hospitalized cohort.

**Number of participants (n)**	196
Mean(SD) Age(years)	41 ± 30
Male gender (%)	58%
**Culture result (n)**	
Negative	177
*Campylobacter*	14
*Campylobacter* & *Salmonella*	1
*Salmonella*	1
*Shigella*	3

### Hospitalized patients have a significant rise in α-diversity (richness within sample) from birth to <4 years of age, which continues up to the second decade of life

**Figure 1:**
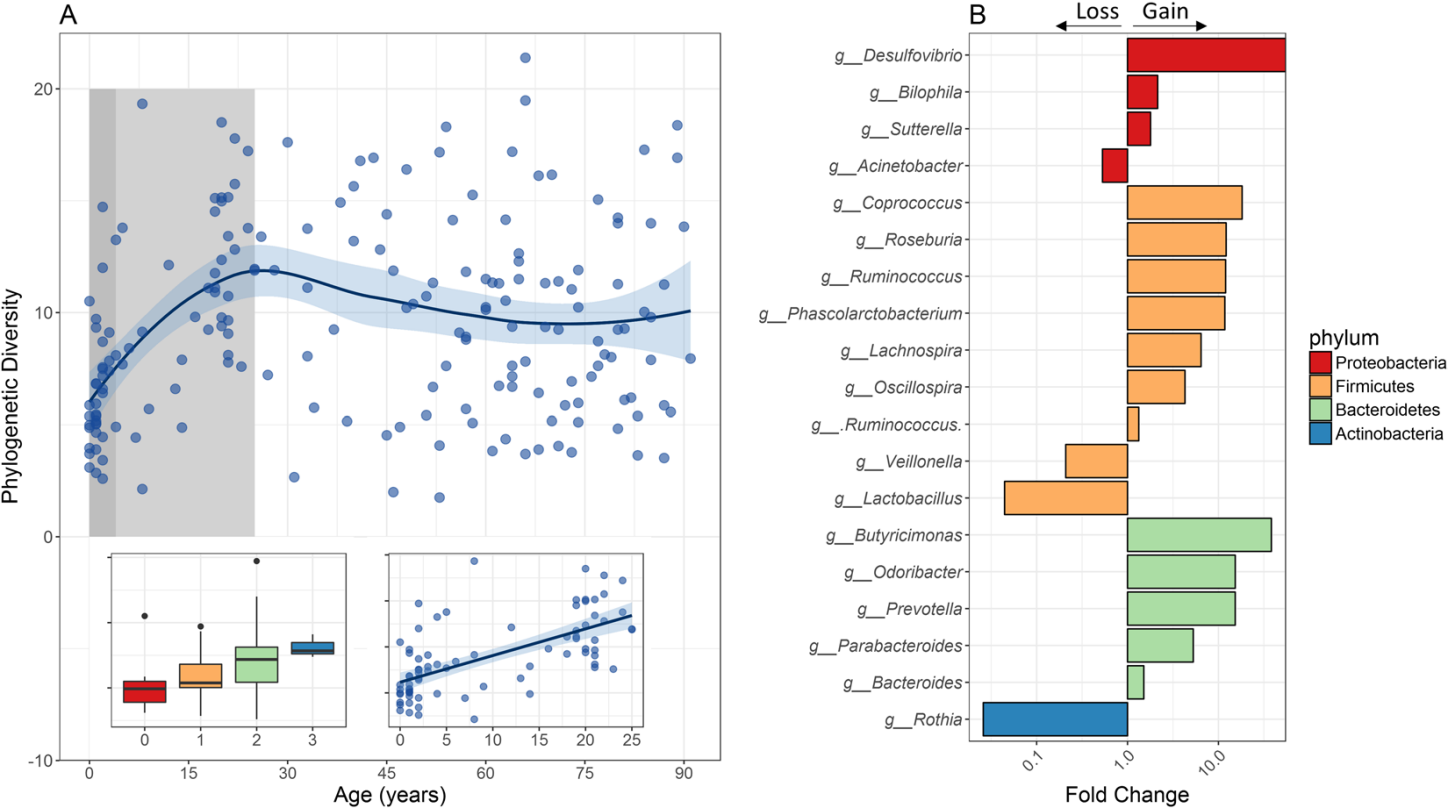
Age-related effect on gut Microbial diversity and composition of hospitalized patients’ with suspected infectious diarrhea. (**A**) Phylogenetic diversity (a measure of within sample diversity, or α-diversity) of hospitalized Israeli patients’ fecal microbiome OTU relative abundance plotted against age, for all sample across all ages, with a local polynomial regression fitting calculated by weighted least squares method, and a 0.95 confidence interval using a t-based approximation. Dark-shaded box represents years 0–4. Light-shaded box represents years 4–25. Right inset represent the same calculation during the first 25 years of life, with a linear regression fitting. Left inset, a boxplot for the first 4 years of life (0–< 4). (**B**) Fold change in taxonomic abundance between adults (18–70 years) and infants and toddlers (0–< 4 years). A fold change greater than one represents a gain in adults, and lower than one a gain in children and toddlers. Results are shown for taxa with a significant change over age, as inferred by a MaAsLin analysis (see methods), at the genus (g) level, for taxa from the phylum Proteobacteria, Firmicutes, Bacteroidetes, and Actinobacteria.

Previous studies have shown that microbial diversity rises during the first years of life^1, 6, 17^. Although hospitalization can be viewed as a perturbation in early life, we noted a significant rise (Spearman’s rank correlation for α-diversity and age for age <4: r = 0.401, *P* = 0.017) in α-diversity (within sample diversity) in the hospitalized patients during the first four years of life (Fig. 1A). However, the α-diversity of the hospitalized population continued to ascend, and only stabilized around the age of 25 (Spearman’s rank correlation for α-diversity and age for age <25: r = 0.672, *P* = 1.206e-11 vs. Spearman’s rank correlation for α-diversity and age for age of ≥25: r = −0.097, *P* = 0.308). To further characterize specific taxonomic changes in the microbiome composition between early and later ages we used Multivariate Analysis by Linear Models (MaAsLin) pipeline, a sparse multivariate association test relying on linear models, which similarly associates the microbiota with multiple, and potentially confounded sample variables^18–20^. We specifically tested for associations within our hospitalized cohort between individuals less than 4 years (0–< 4 years) and adults (18–70 years) while controlling for gender. We were able to identify 43 significant associations between those defined age groups and microbial taxa up to the genus level (*q* < 0.05, Supplemental Table S1). The fold change for specific taxa with significant associations was calculated (Fig. 1B). Young hospitalized patients (0–< 4 years) with suspected infectious diarrhea had a higher abundance of taxa from Actinobacteria phyla, lower abundance of the Bacteroidetes phyla, and a diverse fold change at the genus level within the Firmicutes and Proteobacteria phyla.

### Microbiota from hospitalized patients show significant high abundance of Proteobacteria taxa

**Figure 2:**
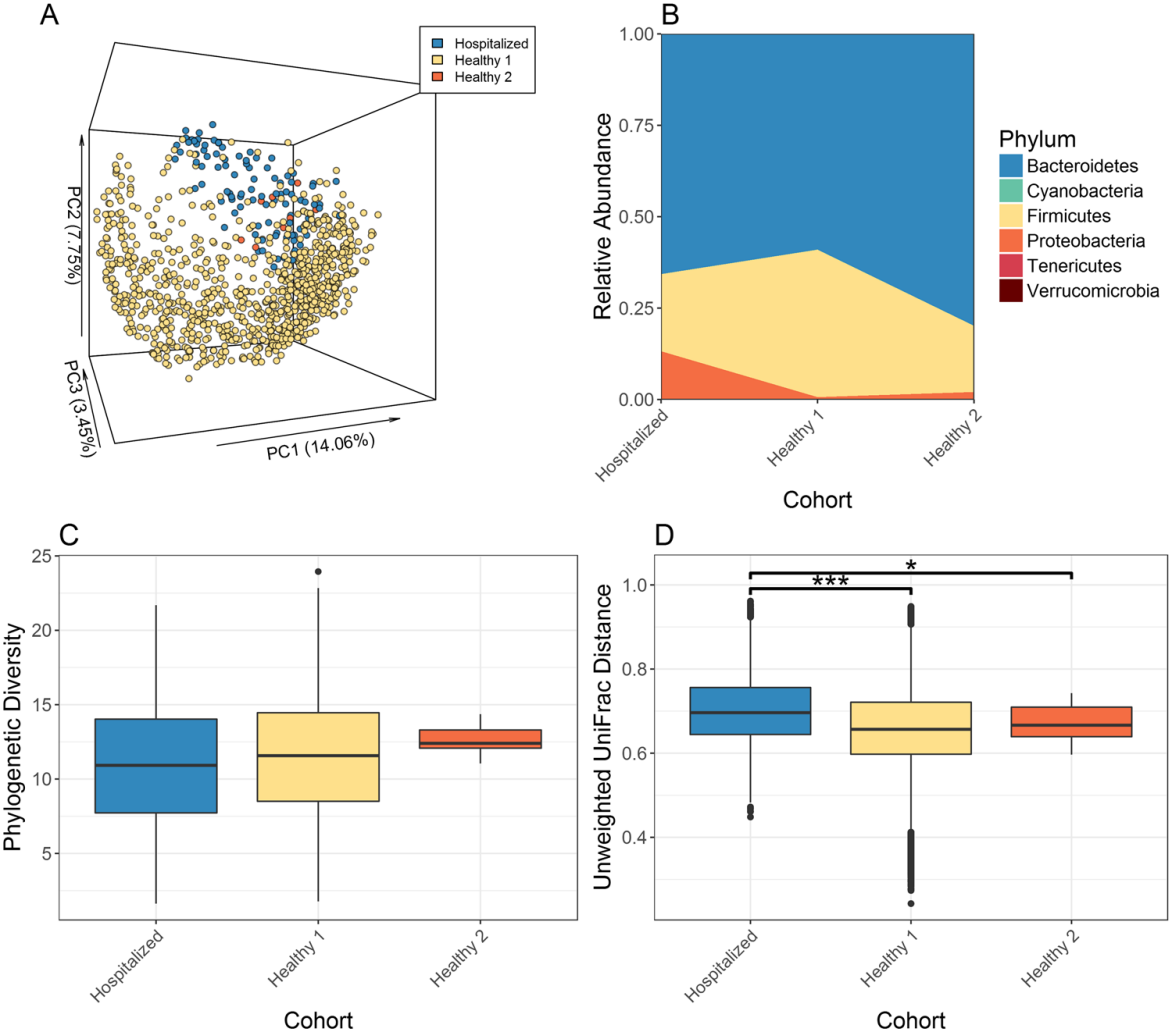
Microbial composition and diversity differences between hospitalized and healthy adults. (**A**) Unweighted UniFrac PCoA plot. Each point represents an adult (18–70 years) gut microbiome sample from one of the cohorts detailed in Table S2. (**B**) Median taxonomic relative abundance at the phylum level by cohort. (**C**) Phylogenetic diversity (within sample diversity) of all adult samples by cohort. (**D**) Unweighted UniFrac distance (between samples diversity) between all samples within a cohort. Asterisks indicate significant differences (Bonferroni corrected Mann Whitney test: ***q < 0.001, *q < 0.05).

To compare the microbiome composition of the hospitalized Israeli patients to that of healthy individuals, we used data from a healthy adult Israeli population^16^ aged 17–80 (Israeli healthy 1), and a local cohort of healthy volunteers (Israeli healthy 2, Supplementary Table S2). Since most samples were from adults (18–< 70 years), and to adjust for age affect, we focused on exploring microbial composition and diversity in adults. To estimate the relationship between the different samples we used unweighted UniFrac^21^ (Fig. 2A) as a measure of β-diversity, or between samples diversity. Principal coordinates analysis (PCoA) that reduces dimensionality of the input data was used on the UniFrac matrix to visually explore sample separation and similarity. Similarities and differences between populations can also be noted when looking at the taxonomic abundance at the phylum level (Fig. 2B). While the stool microbial composition is dominated by the three main phyla: Bacteroidetes, Firimicutes, and Proteobacteria; the median abundance changed between the cohorts. The hospitalized Israeli population showed the highest abundance of Proteobacteria.

α-diversity (Fig. 2C) showed no significant difference between hospitalized Israeli and the healthy Israeli population (by cohort Bonferroni corrected Mann Whitney q > 0.39 between hospitalized and healthy 1 & 2). β-diversity between cohorts (Fig. 2D) showed that hospitalized Israeli samples had significant higher β-diversity than the healthy Israeli population (by cohort Bonferroni corrected Mann Whitney test q < 1.3e-2 between hospitalized and healthy 1 & 2). Interestingly, significantly lower α-diversity was noted in age-matched adult hospitalized patients with positive stool molecular testing for *Clostridium difficile* compared to those with negative testing (Supplementary Fig. S1, *P* = 0.023 for Mann Whitney test between groups).

**Figure 3:**
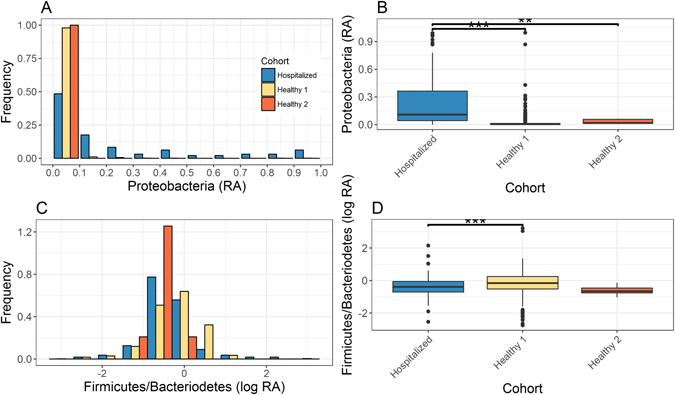
High abundance of Proteobacteria phylum in hospitalized patients. Changes are shown for Proteobacteria (**A**,**B**), and Firmicutes/Bacteroidetes ratio (**C**,**D**). Phylum level taxonomic relative abundance (RA) or log10 of RA ratio histogram (**A**,**C**) and boxplot (**B**,**D**) as indicated. Asterisks indicate significant differences (Bonferroni corrected Mann Whitney test: ***q < 0.001, **q < 0.01).

Israeli hospitalized samples show significantly higher Proteobacteria abundance profiles in comparison to Healthy Israeli population (Fig. 3A and B, Bonferroni corrected compared to hospitalized population *q* = 3.0e-44 for Proteobacteria abundance in Mann Whitney test between hospitalized and healthy 1, and *q* = 2.7e-3 and between hospitalized and healthy 2). In the healthy Israeli population 95% of samples had less than 10% abundance of taxa from the Proteobacteria phylum (Fig. 3A). The hospitalized Israeli population had the highest fraction (>60%) of samples having Proteobacteria abundance above 10%, with abundance >90% in some samples. Since we captured a wide range of Proteobacteria abundance (from under 10% to above 90%) and not just high relative abundance, these results truly reflect the samples actual microbial compositions and are not an artifact of the experimental design. The Firmicutes and Bacteroidetes levels mostly mirror each other, often referred to as the Firmicutes/Bacteroidetes ratio. Previous work has used this ratio to describe associations with increasing age^22^, geography^3^, and obesity^4, 23, 24^. Here, we demonstrate that that hospitalized Israeli show significantly lower ratio then Israeli healthy 1 cohort (Fig. 3B and C Bonferroni corrected compared to hospitalized population q = 7.5e-4 in Mann Whitney test).

### Taxonomic difference between adult cohorts

**Figure 4:**
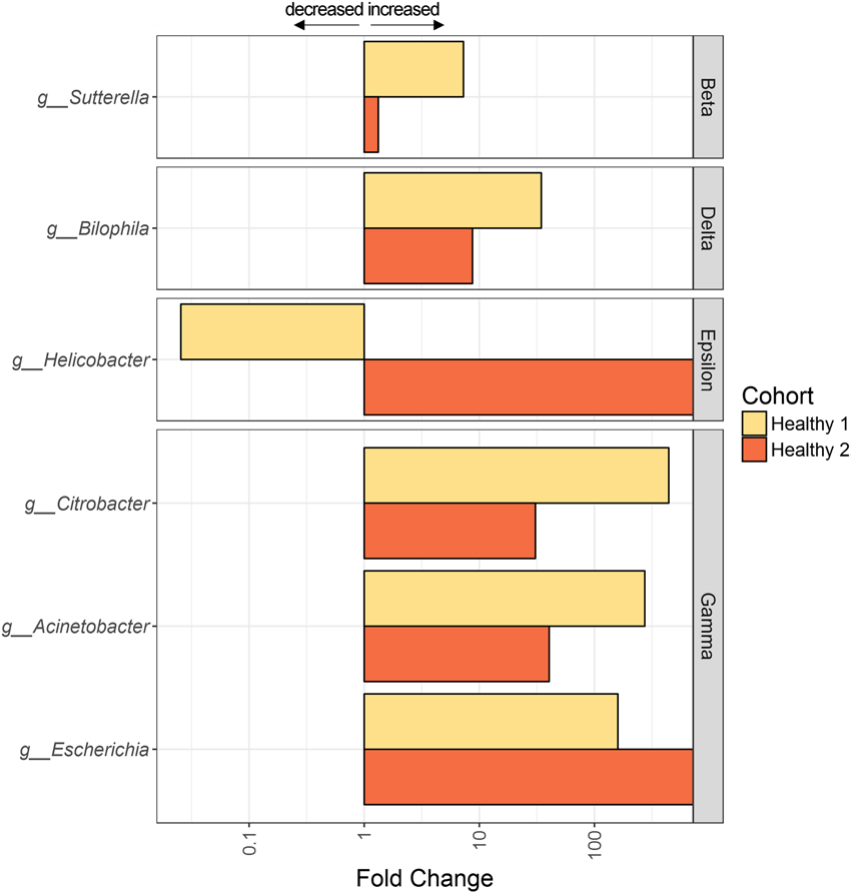
Significant increase of taxa from the Proteobacteria phylum in hospitalized patients. Fold change in taxonomic abundance of the Israel hospitalized cohort, relative to healthy Israeli population (Israeli health 1 & 2) is shown. A fold change greater than one represents an increase in hospitalized Israeli patients, and lower than one a decrease in hospitalized Israeli patients, compared to the other cohorts. Results are shown for significant taxa as inferred by a MaAsLin analysis (see methods), at the genus (g) level for the Betaproteobacteria (Beta), Deltaproteobacteria (Delta), Epsilonproteobacteria (Epsilon), and Gammaproteobacteria (Gamma) classes. Full data can be found in Table S3.

To further characterize specific taxonomic changes in the microbiome composition between Israeli hospitalized and healthy 1 we used MaAsLin pipeline^18–20^. We were able to identify 60 significant associations between those defined groups and microbial taxa up to the genus level (*q* < 0.05, Supplemental Table S3). Fold change of significant associations between the Israel hospitalized cohort, relative to Israeli healthy 1 and Israeli healthy 2 for specific taxa, at the genus level, was calculated for the Proteobacteria taxa (Fig. 4). Using MaAsLin we noted significant increases in taxa from the Gammaproteobacteria class including *Citrobacter*, (*q* = *7.26*e-62) and *Escherichia* (*q* = *0.02*) from the Enterobacteriaceae family, and *Acinetobacter* (*q* = *3.4*e-9) from the Moraxellaceae family. Other significant changes included high *Bilophila* (*q* = *3.3*e-52) from the Desulfovibrionaceae family and *Sutterella* (*q* = *0.04*) from the Alcaligenaceae family. Since this was a multivariate analyses we were under powered to include Israeli healthy 2 in the MaAsLin analysis. However, when we tested the fold change of the Israeli healthy cohorts, relative to the hospitalized population, we noted similar difference with increase abundance of those taxa in hospitalized Israeli. Interestingly, we noted a significant lower *helicobacter* from the Campylobacteraceae family, in hospitalized Israeli cohort in comparison Israeli healthy 1 but higher abundance of *Helicobacter* was noted in Israeli healthy 2.

### High Proteobacteria abundance seen by 16S-seq cannot be explained by positive stool culture results

Above 60% (123 of 196) of hospitalized samples had >10% abundance of Proteobacteria, which include harmless and pathogenic taxa. Since those fecal samples were subjected, in parallel, to conventional microbiological culture and broad-range high throughput 16S rRNA sequencing (16S-seq), we were able to assess which samples were also positive cultures for *Campylobacter, Salmonella*, or *Shigella* as reported by the Microbiology Lab. Of the 196 samples, only 19 (10%) samples had positive cultures including; 14 *Campylobacter*, 1 *Salmonella*, 3 *Shigella*, and one sample that was positive for both *Salmonella* and *Campylobacter* (Table 1). To test the ability of the v4 16S-seq to identify *Campylobacter, Salmonella*, or *Shigella*, we also sequenced isolated bacteria colonies that grew in 18 of the 19 above positive cultures together with the stool samples. The 16S-seq pipeline accurately identified all 14 *Campylobacte*r isolates, with >94% of the sequence being associated with the *Campylobacter* genus. Furthermore, the 16S-seq was able to identify all *Shigella* and *Salmonella* isolates (n = 5) as belonging to the Gammaproteobacteria class (>88% of the sequences), further identification to the genus level was lacking due to high sequence similarities between different Enterobacteria from the Gammaproteobacteria class. Using standard bacterial stool culture methods, enteric infection is conceptualized as a binary state, where the pathogen is either present in the gut or not. Using molecular techniques such as the 16S-seq enable looking at the overall microbial composition and relative abundance of specific microbiota in the examined sample. Since we have already shown that the 16S-seq precisely identify *Campylobacter* isolates, we then tested the relative abundance of *Campylobacter* in the original positive stool samples. Of the 14 samples that were positive for *Campylobacte*r, the relative abundance of *Campylobacter* varied remarkably; 6 samples had ≤1%, 6 samples had >1% but ≤10%, and 2 had >10% relative abundance. Altogether the comparison between traditional microbiology culture and v4 16S-seq showed accurate identification of *Campylobacter* but not *Salmonella* and *Shigella* isolates with a relatively high range of relative abundance of *Campylobacter* in the original stool samples.

## Discussion

Our study focused on characterizing the gut microbiome composition and diversity of hospitalized patients suspected to have infectious diarrhea across all ages in comparison to the healthy population in Israel. To our knowledge this is the largest cohort of hospitalized patients with suspected infectious diarrhea that have had their gut microbiota characterized. Importantly, we show that the hospitalized Israeli population with suspected infectious diarrhea showed a significantly high abundance of Proteobacteria taxa. However, the high abundance of Proteobacteria could not be attributed to positive culture results for *Campylobacter*, *Salmonella*, or *Shigella*. Proteobacteria taxa that were highly enriched in hospitalized patients included *Actinetobater* ^25^, *Bilophila* ^26^, *Citrobacter*, *Escherichia*, and *Sutterella* compared to the healthy population. These results are in agreement with previous studies that showed that non-hospitalized children in developing countries who had been diagnosed with moderate severe diarrhea (MSD) had also low positive culture results. These results may therefore imply that the current list of diarrhea-causing pathogens needs to be further investigated and expanded by molecular and culturing techniques. Another potential implication of this study is the possibility that intestinal dysbiosis of hospitalized patients hamper the protective effect of the gut microbial ecosystem thus making patients more susceptible to the development of healthcare-associated infections (HAIs). Prevention of HAI is one of the top priorities of the Center of Diseases Control and Prevention (CDC), but preventive concepts so far did not addressed the potential contribution of the gut microbiota of hospitalized patients to HAI. Interestingly, a recent study showed that in a randomized, double-blind, placebo-controlled trial in hospitalized children, an intake of a mixture containing Lactobacillus GG and micronutrients reduced the incidence of HAI, supporting the hypothesis that certain manipulations of the intestinal microbiota may provide a safeguarding strategy to prevent such infections^15^.

We aimed to characterize the overall hospitalized population and therefore did not use any selection or exclusion criteria. Our study does not take into account many factors that have been shown to affect microbial composition, such as underlying diagnoses, existing co-morbidities, medications, diet, intestinal transit time, patients’ genetics, ethnicity etc. Similar to other human studies, we put these variables into a “black box” that were folded into the analyses. Despite these limitations, we were able to capture significant microbial maturation across early ages in the hospitalized population and a significantly high relative abundance of Proteobacteria taxa that cannot be explained by culture results. Future studies will look more narrowly at inclusion cohorts to determine how specific clinical data, underlying diagnoses, and medication exposure will enable a better understanding of the interactions between specific patterns in the microbiome composition and these important variables.

In our study we have noted other expected limitations regarding to the inability of the 16S-seq to specifically identify enteric pathogens (*Salmonella* and *Shigella)* and the limitation of traditional culture methods to inform about relative abundance of pathogen rather than positive/negative results as in the cases of positive *Campylobacter*. As previously noted^27–29^, sequencing technologies have gained popularity in clinical microbiology applications but read lengths limits their usefulness for high-resolution taxonomic identifications. On the other hand advantages of using sequencing include identifications of difficult to culture organisms or cases of polymicrobial infections^30^. The use of shotgun metagenomic sequencing may overcome some of those limitations associated with more accurate identification at the species level. As already suggested a potential way to improve identification using 16S-seq is to use well-annotated database of 16S-seq^27^ that improves genus-level identification. We have deposited the 18 isolates that were sequenced in our study in the public domain. It is possible that future microbial testing will benefit from combining traditional culturing with 16S-seq or metagenomic sequencing to enforce more accurate identifications.

In agreement with previous studies, we noted gut microbiome maturation during the first years of life^1, 6, 17^. However, more uniquely, we found that hospitalized gut microbiome did not stabilize by the age of three and α-diversity kept ascending until the second decade of life. This slower maturation could be attributed to medical condition or treatments^17, 31–34^. However, these factors do not seem to affect older patients as there was no significant difference between hospitalized and healthy Israeli adult α-diversity. Still, it is possible that this effect is stronger during early life, before the child’s microbiome fully matures, resulting in the slower maturation of the gut microbiome. It is possible that the slower microbiome maturation is geography related, a possibility that will be explored in future studies of healthy Israeli pediatric population. Despite the slower microbiome maturation, the specific bacteria that are significantly different between infant and toddlers (0–4 years) and adults (18–70 years) are mostly consistent with those found in healthy populations. Specifically, the younger hospitalized patients had a higher abundance of Actinobacteria^35, 36^ that has been associated with breakdown of human milk oligosaccharides^6^. They also had a lower abundance of Bacteroidetes^2, 22^ including *Prevotella* ^1, 3, 14^, and an increase of taxa from the *Lactobacillales* order and the *Gammaproteobacteria* class^1, 37, 38^ in younger patients.

Altogether, understanding microbiome abundance and diversity, as another aspect of capturing difference and similarities between individuals has been the focus in recent years in healthy and pathological conditions. It has been shown that age, geography, lifestyle, and illness are associated with the gut microbiome composition, but many populations and conditions have not yet been fully characterized. To our knowledge, our study is the largest to characterize the gut microbiome of non-selected hospitalized patients with suspected infectious diarrhea. We show that hospitalized patients with suspected infectious diarrhea have significant high abundance in Proteobacteria taxa. Hospitalized patients are already at increased risk for acquiring HAIs and compromised nutritional status. Characterizing hospitalized patients microbiota is the first step for consideration of nutritional or other interventions to restore those alterations and provide better protection against some of those pathogens.

## Methods

### Samples collection and study design

Stool specimens from hospitalized individuals with suspected infectious diarrhea were prospectively subjected, in parallel, to conventional microbiological culture performed at the Clinical Microbiology Lab at the Sheba Medical Center in Israel and broad-range high throughput 16S rRNA amplicon sequencing (16S-seq). No selection or exclusion criteria were applied for sample analysis other than excluding duplicate samples collected more than once from the same individual. In such cases, where multiple samples were collected, only the initial sample was included in the analyses. 8 additional healthy Israeli stool samples were included as controls. Sterile swabs were used to collect stool from stool samples submitted to the lab after processing those samples for routine culturing. Sterile swabs were put in sterile tubes and were placed in −80 °C within 24 hours of stool submission. In addition to the collected stool samples, 19 isolated colonies obtained from those 196 samples including 14 *Campylobacter*, 2 *Salmonella*, and 3 *Shigella*, were collected and frozen in −80 °C using the same sterile swabs. Negative controls including swab blanks (sterile swabs), extraction blanks (reagents), and PCR controls were also included in the sampling and analyses. 196 unique stool specimens from hospitalized individuals with suspected infectious diarrhea and 8 healthy controls passed processing, quality control, and filtering. These were included in this study. Ethical approval for the study was granted by the Sheba Local Research Ethics Committee and all methods were performed in accordance with the relevant guidelines and regulations. Since we used fecal material already submitted to the microbiology core as part of clinical workup and without identifiable patient information other than age, gender, and microbial results, we got exception from patients consent from the local Ethical Review Board. Healthy individual’s data were obtained from publicly available databases^16^ (Israeli healthy 1). In this study our primary goal was to characterize the microbial composition and diversity of non-selected Israeli hospitalized individuals with suspected infectious diarrhea in comparison to healthy population. Our secondary goal was to test how 16S-seq results correlates with traditional culture results.

### DNA extraction, PCR amplification, and sequencing

DNA extraction and PCR amplification of the variable region 4 (V4) of the 16S rRNA gene using Illumina adapted universal primers 515F/806R^39^ was conducted using the direct PCR protocol [Extract-N-Amp Plant PCR kit (Sigma-Aldrich, Inc.)] as previously described^40, 41^. Aliquots (4 μL) from the stool extracts were transferred into 96-well plates. PCRs were conducted in triplicate 20 μL reactions and thermal cycling conditions were: initial denaturation for 3 min at 94 °C; 35 cycles (98 °C, 60 s; 55 °C, 60 s; 72 °C, 60 s) followed by a final elongation for 10 min at 72 °C. PCR products from triplicate reactions of each sample were pooled, visualized on an agarose gel, and quantified using the PicoGreen dsDNA assay (Invitrogen, Carlsbad, CA, USA). Positive amplicons were then pooled in equimolar concentrations into a composite sample that was size selected (300–500 bp) using agarose gel to reduce non-specific amplification products from host DNA. A final library size and quantification was done on an Agilent Bioanalyzer 2100 DNA 1000 chips (Agilent Technologies, Santa Clara, CA). Sequencing was performed on the Illumina MiSeq platform at the Cancer Research Center at the Sheba Medical Center. We used the v3 kit, according to the manufacturer’s specifications with addition of 20% PhiX, and generating paired-end reads of 175b in length in each direction.

### Data processing

The overlapping paired-end reads were stitched together and further processed in a data curation pipeline implemented in QIIME 1.9.1 (QIIME allows analysis of high-throughput community sequencing data). Paired-end reads with an overlap of less than 50 nucleotides, or more than 5% difference within the overlap region, were excluded. The joined reads were further quality controlled by truncating reads at nucleotides with a Phred score lower than 30. Reads that had ambiguous characters, or were truncated to less than 75% of the original length, were discarded. The remaining reads were binned according to sample specific barcode. No barcode errors were tolerated. Sequences that passed quality filtering were clustered into phylotypes (Operational Taxonomic Units, OTUs) at 97% sequence identity using a uclust-based^42^ closed-reference protocol against the October 2012 revision of the Greengenes database^5^, where reads that did not match a sequence in the reference set at least 97% identity were excluded from subsequent analyses. The taxonomy of each phylotype was assigned as the taxonomy associated with the Greengenes sequence defining that OTU (Supplemental Table S4). The Greengenes phylogenetic tree was used for phylogenetic diversity calculations. Negative control samples had an average of 17 sequences primarily from the Mycoplasma, where all samples analyzed showed an average of 0.6323 reads belonging to those taxa (maximum of 10 reads per sample). These taxa were removed from the OTU table. A median of 5,553 (average of 6,240) paired end sequences were collected per sample. As the Israeli healthy adult data^16^ included repeating samples from some individuals, samples with a UniFrac^21^ score under 0.05 similarity to other samples within the dataset were removed. All samples were rarefied to 1,000 sequences for downstream α and β diversity analysis and relative abundance was used, to avoid sample size affect. Phylogenetic diversity (PD)^43^ was used as a measure of α-diversity = within samples diversity. Unweighted and weighted UniFrac was used as a measure of β-diversity = between sample diversity^21, 44^. The resulting distance matrix was used to perform a PCoA analysis.

### Taxonomy Abundance differences and fold change

Abundance at different taxonomy levels was calculated using the QIIME scripts summarize_taxa.py.

The huttenhower galaxy version of MaAsLin version 1.0.1 with default parameters was used to identify taxa that had a significant change between different groups while controlling for other variables. MaAsLin is a multivariate statistical framework that finds associations between clinical metadata and microbial community abundance or function. Taxa were tested only up to the genus level and taxa that appeared (at least one read in the sample) in less than 10% of the samples were removed. MaAsLin analysis was performed for younger (<4) versus older (18–70) ages within the hospitalized Israeli population while controlling for gender, and for Israeli hospitalized and Israeli healthy 1 while controlling for gender and age group (0–4, 4–18, 18–70 and 70+). Fold change was calculated at the genus level for taxa that had a q value lower than 0.05. Fold change between two groups was calculated by dividing the mean abundance of taxa in those groups.

## Declarations

### Ethics approval

Ethical approval for the study was granted by the Sheba Local Research Ethics Committee and all methods were performed in accordance with the relevant guidelines and regulations. Since we used fecal material already submitted to the microbiology core as part of clinical workup and without identifiable patient’s information other than age, gender and microbial results, we got exception from patients consent from the local Ethical Review Board. Written, informed consent was obtained from healthy volunteers and the Institutional Review Board approved the study.

## Electronic supplementary material

Supplementary info

Supplementary Table S1

Supplementary Table S3

Supplementary Table S4
