# Supplementary material for: Fecal microbial characterization of hospitalized patients with suspected infectious diarrhea shows significant dysbiosis
Source: Sci Rep. 2017 Apr 24;7:1088. doi: 10.1038/s41598-017-01217-1 (PMC5430810; doi:10.1038/s41598-017-01217-1)
Supplement: Supplementary file 1 — Supplementary info [file 41598_2017_1217_MOESM1_ESM.pdf]

Supplementary Information

Fecal microbial characterization of hospitalized patients with suspected infectious diarrhea shows significant dysbiosis

Tzipi Braun*[1], Ayelet Di Segni*[1], Marina Ben Shoshan[1], Roy Asaf[1], James E. Squires[2], Sarit Farage Barhom[1], Efrat Glick Saar[1], Karen Cesarkas[1], Gill Smollan[1], Batia Weiss[1,3], Sharon Amit[1], Nathan Keller[1,3], Yael Haberman[1,4]

[1]Sheba Medical Center, Tel-HaShomer, 5265601, Israel

[2]Children's Hospital of Pittsburgh of UPMC, 4401 Penn Ave, Pittsburgh, PA, 15224, USA

[3]Tel Aviv University, Ramat Aviv, Tel-Aviv, 6997801, Israel

[4]Cincinnati Children's Hospital Medical Center, 3333 Burnet Ave, Cincinnati, OH, 45229, USA.

*equal contribution

Corresponding Author:

Yael Haberman, MD, PhD

Division of Pediatric Gastroenterology, Hepatology & Nutrition

Cincinnati Children's Hospital Medical Center

MLC 2010, 3333 Burnet Avenue

Cincinnati, OH 45229

Yael.haberman@cchmc.org

List of suppl. Figures

Figure S1. α-diversity decreases in patients with *Clostridium difficile*.

List of Suppl. Tables

Table S1 (separate Excel file). MaAsLin analysis results for Israeli hospitalized samples stratified by ages 0-<4 years and 18-70 years and microbial abundance.

Table S2. Demographic characteristics of the cohorts.

Table S3 (separate Excel file). MaAsLin analysis results for comparison between cohorts, and microbial abundance.

Table S4 (separate Excel file). Relative abundance OTU table of Israel hospitalized cohort, isolates and controls.

Figure S1: α-diversity decreases in patients with *Clostridium difficile*.

Phylogenetic diversity of samples from patients diagnosed for *Clostridium difficile* infection (n=8 patients). Negative samples are age-fitted to samples with a positive diagnosis (n=68 patients, aged 39-91 years). Asterisk indicates significant differences (Mann Whitney test: * $P < 0.05$).

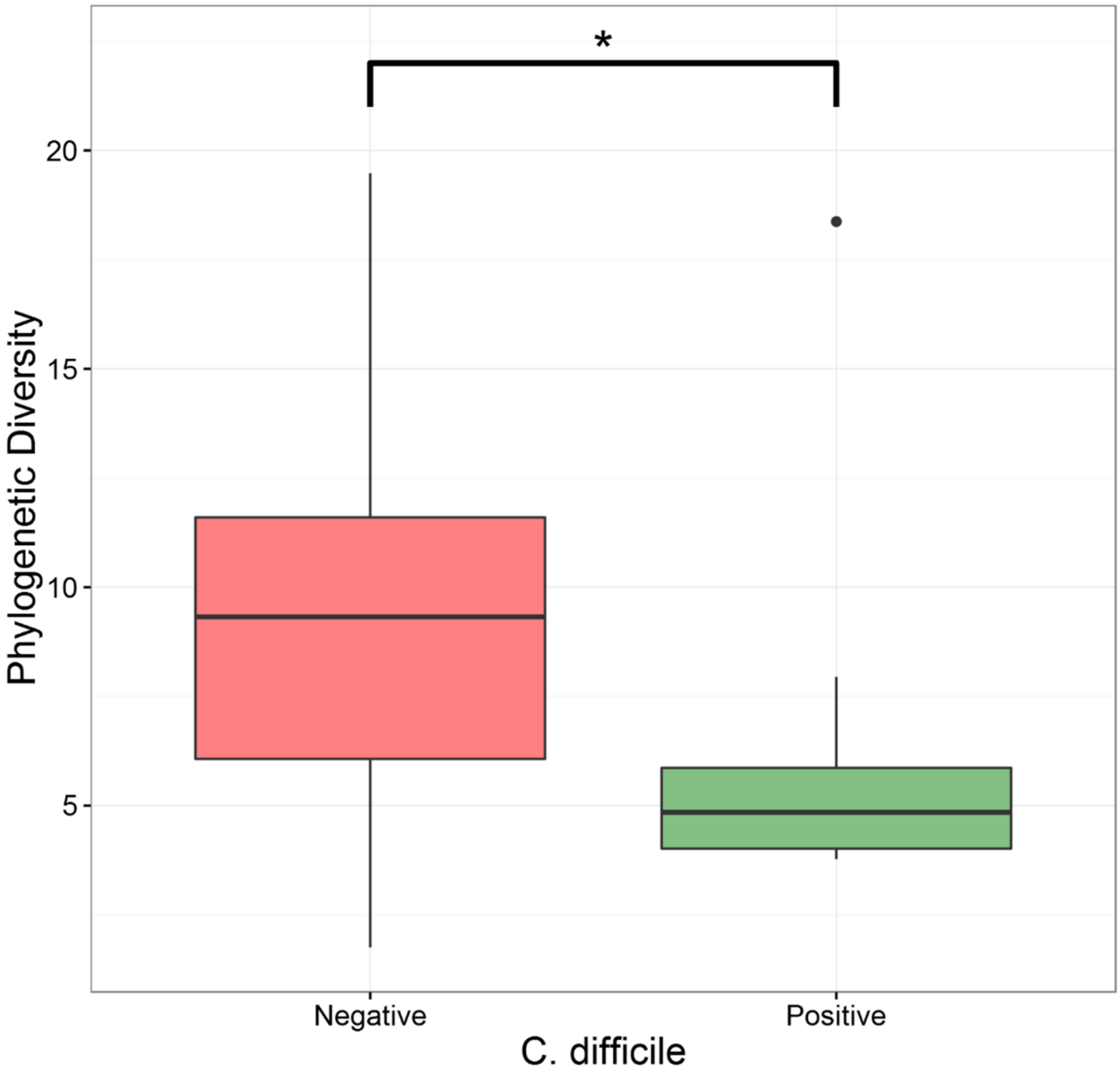

Table S2: Demographic characteristics of the cohorts

Cohort	No. of participants (n)	Mean(SD) Age(years)	No. of infants and toddlers (<4years) (n)	No. of children (4-<18years) (n)	No. of adults (18-<70years) [n, mean(SD) age(years)]	No. of elders (≥70years) (n)
**Israeli hospitalized**	196	40.8 ± 29.8	35	16	97 (43.5± 17.8)	42
**Israeli healthy 1**	873	43.3 ± 13.1	0	0	873 (43.3 ± 13.1)	0
**Israeli healthy 2**	8	51 ± 9.7	0	0	8 (51 ± 9.7)	0
